# Chitosan–organosilica hybrid decorated with silver nanoparticles for antimicrobial wearable cotton fabrics

**DOI:** 10.1007/s00289-022-04250-x

**Published:** 2022-05-17

**Authors:** Lamis Ahmed Aboelmagd, Emad Tolba, Zeinab Ahmed AbdelAziz

**Affiliations:** 1 Home Economics Department, Faculty of Specific Education, Mansoura University, Mansoura, Egypt; 2 Polymers and Pigments Department, National Research Centre, 33 El Bohouth St., Dokki, P.O. BOX 12622, Giza, Egypt

**Keywords:** Silver nanoparticles, Functional cotton fabrics, Antibacterial activity, Chitosan–organosilica hybrid, Nanostructured materials

## Abstract

Functional cotton fabrics using silver-based nanoparticles (AgNPs) have attracted a lot of attention as a new generation of healthcare wearable textile. In this study, cotton fabrics were coated via impregnation with silver nanoparticles using chitosan (Cs) and (or) chitosan–organosilica (Cs-OSH) solutions as adhesives matrices. The physicochemical properties were studied using UV–VIS spectroscopy, and transmission electron microscopy (TEM) and scanning electron microscope coupled with energy-dispersive X-ray spectroscopy methods (SEM–EDX). The antibacterial activity of the silver-treated fabrics was determined using agar diffusion method. However, nanosize spherical AgNPs were observed in Cs and Cs-OSH solution. The average particle diameter was around 10 nm for Cs/AgNPs sample and close 21 nm for Cs-OSH/AgNPs. Microscopy images showed the deposition of Ag NPs on the surface of cotton fibers. The results indicated that the cotton fibers treated with Cs-OSH/AgNPs solution showed good stability against washing and maintained higher antimicrobial activity even after being exposed to 10 consecutive home laundering conditions. Thus, this work suggests the use of chitosan–organosilicon matrix to improve the bonding between AgNPs and cotton fibers for better and long-term antimicrobial activity.

## Introduction

A significant growth has been observed in the development of multifunctional fabrics, especially antibacterial ones, to replace currently used natural or synthetic textile materials, including cotton, wool, silk, nylon, polyester and polyamide, which are known to provide suitable environments for pathogenic microorganisms growth and diseases spreading, because of their surface features and moisture absorptions [1, 2]. Therefore, there have been much efforts to face the demands and to fight against bacterial infections by using nanosize metal-based particles as antimicrobial agents to bring new functional properties for finishing of institutional textile domains including hospital clothes and other surgical tools (i.e., caps, gowns and drapes) [3, 4]. Metal-based nanoparticles including silver (AgNPs), gold (AuNPs), zinc oxide (ZnONPs), titanium oxide (TiO_2_NP), copper (CuNPs), cuprous oxide (Cu_2_O) and copper oxide (CuONPs) have been shown to exhibit remarkable strong and sustainable antimicrobial activity against a wide array of bacteria, fungi, algae and even virus [5–9]. The metal-based nanoparticles involve distinct multiple bactericidal mechanisms including oxidative stress, interaction with bacterial components biomolecules(damage of cell membrane or cellular organelles) and non-specific mechanism (i.e., disruption of cell signaling process), which evidently underline the powerful potential of nanosize metal-based particles as effective alternative antibacterial agents to avoid bacteria antibiotic-resistance mechanism [10, 11].

Inspired by the special biological features of silver nanoparticles (Ag NPs) such as antibacterial [11], antiviral [12] and anti-inflammatory activities [13], fabrication of silver nanoparticles-coated cotton fabric has attracted considerable interest in biomedical application, as wound dressing to accurate healing process and fight against bacterial infections [14, 15]. However, the synthesis of nanosized metallic particles has been carried out using three different approaches, including physical, chemical and biological methods [14]. Physical methods (i.e., evaporation–condensation and laser ablation) and chemical routes using reducing agents including sodium citrate and sodium borohydride (NaBH_4_) seem to be more expensive, risky and complicated due to the reducing chemical agents involved in reduction of silver ions to silver nanoparticles [14, 15]. On the other hand, biologically synthesized AgNPs show high yield and better colloidal stability [16].

Chitosan is a natural linear polysaccharide composed of randomly distributed *β*-(1–4)-linked D-glucosamine and N-acetyl-d-glucosamine units [17]. Chitosan is derived from chitin which forms the exoskeleton of many living organisms, including shrimps, crabs, insects and cell walls of fungi [18, 19]. Chitosan has favorable physicochemical and even biological properties, such as biodegradability, biocompatibility, bioactivity, high permeability and non-toxicity [18, 20]. As a bioactive polymer, chitosan-based materials reveal better dissolution, degradation and antimicrobial activity over cellulose due to the protonation of the amino groups along its backbone in acidic conditions. The amino groups are able to interact with negatively charged components of microbial membranes, resulting in the bacterial death through changing the permeability of bacterial cell membrane [21].

Silicate-based materials have attracted extensive attention and research interest for biomedical application due to their high biocompatibility features, mesoporous structure and surface reactivity [22, 23]. Although silica nanoparticles [SiO_2_NP] have been applied for the controlled release of therapeutic molecules and also metal nanoparticles [25]. The intrinsic stability of silica NPs stemming from the high stability of − Si–O–Si − framework remains a challenge [26]. An emerging trend is to produce multifunctional silicate-based materials by introducing organic terminals into the silica framework to change the internal framework of silica NPs and promote their physiological biodegradation potential which gives birth to a new type of molecularly organic − inorganic hybrid of organosilica structures [27, 28].

Hereby, the objective of the present study was planned to chemically synthesize a colloidal solution of chitosan–organosilica hybrid system decorated with silver nanoparticles to improve the chemical affinity between the cellulosic chains and the fabricated Ag nanoparticles. Morphological microstructure of the fabricated AgNPs was assessed using TEM and SEM. Consequently, after several repeated washing steps, the antibacterial potential of the processed cotton fabric was also examined.

## Materials and method

Low molecular weight Ch (89% degree of deacetylation), ascorbic acid (vitamin C) triethoxy(octyl)silane and silver nitrate (AgNO_3_) were purchased from Sigma-Aldrich Co. (St. Louis, MO, USA). Cotton fabric (CO—100%, 147 g/m^2^) from El-Mahala for Textile Industry Co., Egypt, was used.

### Preparation of Ag colloidal solutions

A solution of chitosan (1% w/v) in 0.5% acetic acid solution was firstly prepared at room temperature. The obtained solution was centrifuged at 5000 rpm for 10 min to remove insoluble contents. About 10 mL of freshly prepared AgNO_3_ (0.1 M) was added to 100 ml chitosan solution. The solution was kept under stirring for 10 min at room temperature. Then, 1 ml of ascorbic acid (0.1 M) was slowly added to the chitosan/AgNO_3_ mixture. The color of the solution was changed from colorless to light yellow and finally to yellowish brown indicating the formation of Ag NPs. In order to prepare chitosan–organosilica hybrid decorated with silver nanoparticles (Cs-OSH/Ag NPs), triethoxy(octyl)silane was added to Cs before AgNO_3_ addition. In detail, 0.5 ml of triethoxy(octyl)silane was diluted with 5 ml ethanol and slowly added into Cs solution. The chitosan–organosilica solution was kept under mild stirring for 1 h. Afterward, silver nitrate and ascorbic acid were subsequently added as mention above. All the reactions were performed away from light to prevent oxidation of silver ions.

### Cotton fabric treatment with silver colloidal solutions

The cotton fabrics were washed in ultrasonic water bath containing 4% of sodium lauryl sulfate for 10 min at 40 °C and then washed three times distilled water, before air drying at 40 °C overnight. The dried fabrics were immersed into to the Cs/Ag NPs (or Cs-OSi/Ag NPs) solution and then gradually heated to 100 °C (2 degree per 1 min) for 1 h. After that, the fabric was removed and washed with water before immersion in sodium hydroxide solution (0.1 M) for 5 min to stabilize of Cs/Ag NPs on top of the cotton fibers. Finally, the cotton fabrics were rinsed with distilled water and dried for 10 min 100 °C.

### Characterization of Ag NPs

#### UV–visible spectral analysis

Synthesized Ag NPs absorption peak was observed in the UV–visible spectrophotometer (Shimadzu (UV 2500), Japan). UV–visible spectrophotometer range was from 200 to 900 nm.

#### Microscopic analysis

The TEM technique was employed to visualize the morphology and the size of the synthesized Ag NPs using Ultra High Resolution Transmission Electron Microscope (JEOL-2010). TEM grids were prepared by placing a drop of the particle solution on a carbon-coated copper grid and drying under lamp. In addition, the cotton treated samples were coated with gold and examined using field emission scanning electron microscope (SEM) (Jeol JXA 840, Japan).

#### Silver content

The silver-treated samples were digested in concentrated nitric acid solution using Anton-Paar microwave digestion system (Multiwave PRO) (29). The determination of silver ions was conducted using the Agilent 5100 Synchronous Vertical Dual View (SVDV) inductively coupled plasma-optical emission spectrometry (ICP-OES), with Agilent Vapor Generation Accessory VGA 77. Accuracy and precision of the silver ions contents were confirmed using external reference standards from Merck, and standard reference material and quality control sample from National Institute of Standards and Technology (NIST) were used to confirm the instrument reading.

#### Washing stability tests

To investigate the washing stability performance of the cotton fabric treatment with silver colloidal solutions, the samples were washed in a domestic washing machine. The treated cotton samples were washed with tap water containing 4% of sodium lauryl sulfate at 40 °C for 30 min. The washed samples were next dried at room temperature. This process was then repeated for each sample for 10 times. The surface morphology of washed cotton fabrics after washing was investigated by scanning electron microscope.

## Antimicrobial activity

### Minimum inhibitory concentration (MIC)

Antimicrobial activity of the as-prepared samples was assessed against three different microorganisms including gram-negative bacteria (*Escherichia coli*: ATCC25922), gram-positive bacteria (*Bacillus subtilis*: ATCC6633) and unicellular fungi (*Candida albicans*: ATCC90028) [30–32]. Bacterial strains were cultured on nutrient agar at 37 °C for 24 h, while fungal strains were seeded on malt extract agar (MEA) plates, then incubated for 3 days at 28 ± 2 °C and then kept at 4 °C for further use.

The minimal inhibitory concentration (MIC) was determined for Cs/AgNPs and Cs-OSH/AgNPs colloidal solution according to the M07-A9 protocol of the Clinical Laboratory Standards Institute [30]. The as-prepared silver-containing solutions were diluted to different concentrations ranged from 1000 to 10 µg/mL and assessed separately to detect MIC against selected bacterial (using Amoxicillin Clavulanate (AMC) as a standard antibiotic) and fungal strains (nystatin as a standard antifungal agent [31].

### Agar well diffusion method

Antimicrobial activity of the prepared colloidal and cotton fabric samples was assessed using agar well diffusion method to determine inhibition zone of tested samples against different isolated bacterial and fungal pathogens [30, 32]. The bacterial suspensions of 1.5 × 10^6^ CFU/mL were separately prepared and seeded into sterilized petri plates of nutrient agar. 100 µL of the tested Cs/AgNPs and Cs-OSH/AgNPs samples at concentration 200 mg/mL was added in agar well, and then plates were kept in refrigerator for 2 h followed by incubation at 37 °C for 24 h. On the other hand, the fungal suspension was prepared in sterilized phosphate buffer solution (PBS) pH 7.0, and then the inoculums was adjusted to 10^7^ spores/mL after counting in a cell counter chamber. One mL was uniformly distributed on agar MEA Plates. Sterile cork borer (75 mm) was used for making well in inoculated MEA plates, and then 100 µl of the tested samples was added. The obtained MEA plates were incubated at 30 °C for 72 h, and then the inhibition zone diameter was measured.

## Results

### Microstructure analysis of as-prepared AgNPs colloidal solutions

**Fig. 1:**
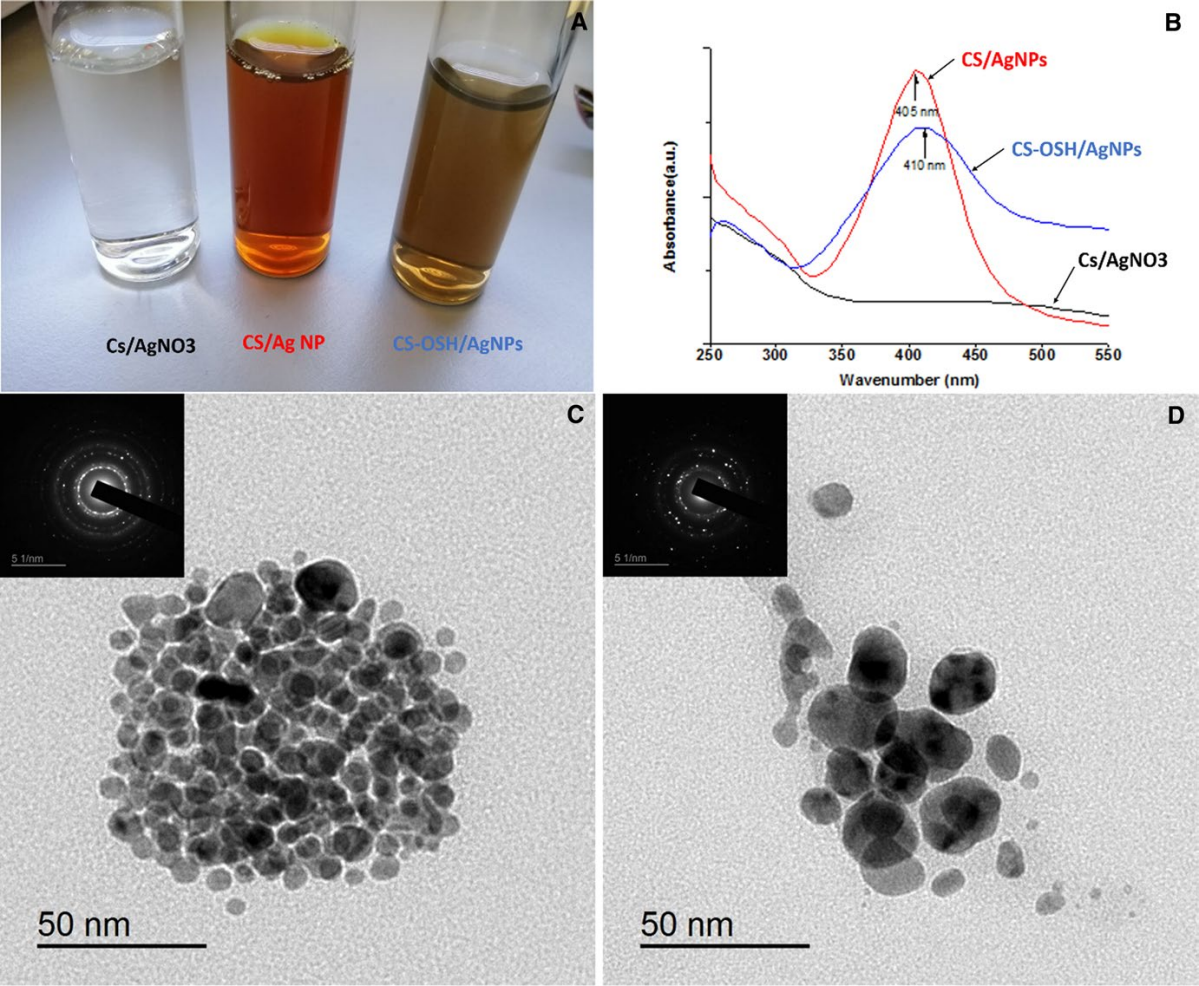
Characterization of the fabricated AgNPs colloidal solutions: **A** Color change of Cs/AgNO_3_ solution after the addition of ascorbic acid, **B** UV–visible spectrophotometer range was from 250 to 550 nm, **C** TEM image of Cs/Ag NP solution. Inset shows selected area electron diffraction (SAED) pattern of silver nanoparticles **D** TEM image of Cs-OSi/Ag NP colloidal solution and SAED pattern

**Fig. 2:**
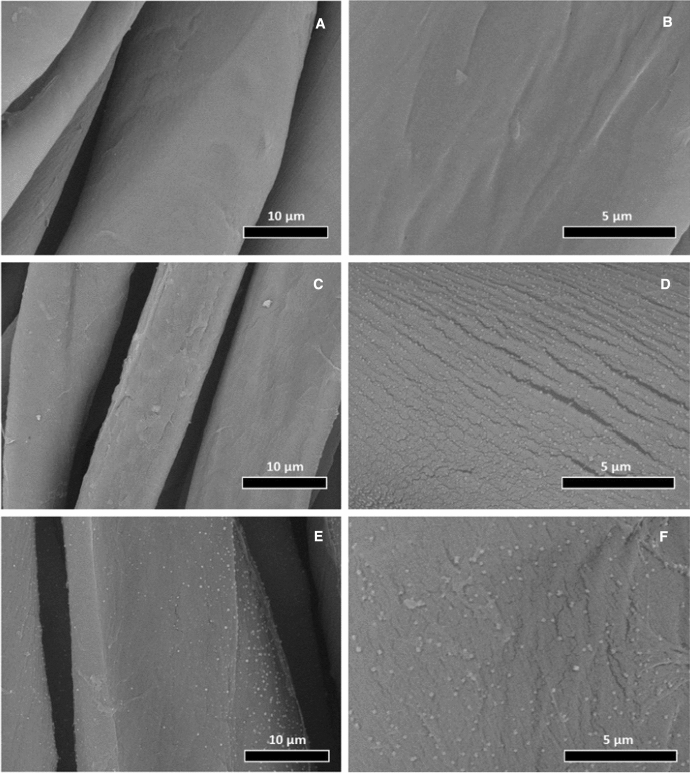
SEM micrographs of the (**A**, **B**) cotton fabric, **C**, **D** cotton-Cs/AgNPs sample and **E**, **F** cotton-Cs-OSH/AgNPs sample

As a natural polysaccharide, chitosan has been used as a capping agent to prevent aggregation and to improve colloidal stability of metallic nanoparticles including silver and gold nanoparticles [33–36]. As shown in Fig. 1, the formation of the AgNPs after the reduction process was clearly observed by change in the color of the Cs solution from colorless to yellowish brown for CS-AgNPs and dark brown for CS-OSH/AgNPs solutions (Fig. 1-A). The as-prepared colloidal solutions display strong UV–vis absorption in the visible region which is attributed to the surface plasmon resonance (SPR) of AgNPs. The UV–Vis spectra show a gradual decrease of the absorbance for CS-OSH/AgNPs solution (blue colored spectrum) compared to CS-AgNPs solution (red colored spectrum) and also a slight shift at the max wavelength from 410 to 405 nm was observed, as shown in Fig. 1-B. However, the presence of a single SPR peak confirms the formation of monodispersed spherical Ag NPs within Cs matrix. The slight shift and color changes among the samples are due to the variation in the size of the AgNPs. TEM images of AgNPs, synthesized in Cs and Cs-OSH solutions, are presented in Fig. (2-c and d). As can be seen in both images, the AgNPs presented spherical shape. In addition, some oval- and triangle-shaped nanoparticles were observed. The obtained TEM results indicated that the AgNPs diameter for chitosan (Cs) solutions was different from the AgNPs formed in the Cs-OSH solution. The average diameter of Cs/AgNPs was around 9 ± 3 nm, which increased to 21 ± 6 nm in Cs-OSH/AgNPs. The inserted SAED images reveal a diffraction pattern indicating crystalline structure of prepared AgNPs.

The surface morphology of the cotton fabrics samples was investigated by SEM as shown in Fig. 2. Images of raw cotton fabric (Fig. 2a and b) show smooth longitudinal fiber structure without any contaminating particles on their surfaces. The cotton fabrics treated with Cs/Ag NPs fabrics were covered with ultrafine silver nanoparticles (Fig. 2c and d). In the same context, the images of cotton fabrics treated with Cs-OSH/AgNPs demonstrated a rougher surface with a hierarchical structure due to the deposition of Cs-OSH/AgNPs on cotton fabric, as shown in Fig. 2e and f. Compared to TEM images, the SEM images indicate agglomeration of AgNP during the treatment of cotton fabrics; thereby, the deposited AgNPs appeared larger [5, 7].

**Fig. 3:**
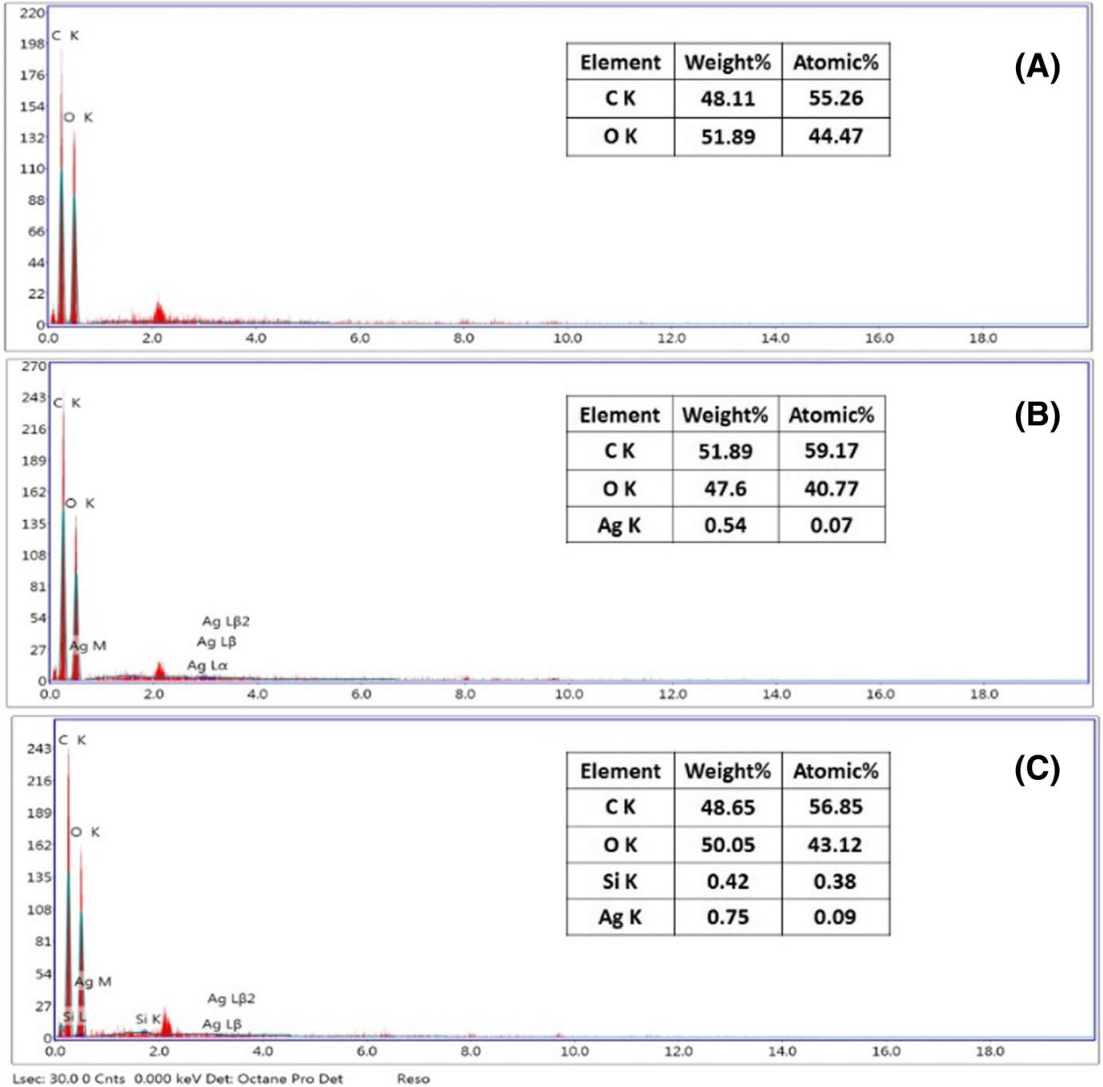
EDX analysis of the **A** cotton fabric, **B** cotton-Cs/Ag NPs sample and **C** cotton-Cs-OSH/Ag NPs sample

Surface chemical elements of the fabrics after and before treatment were determined by EDX spectroscopy. Figure 3 shows EDS spectra of the cotton and cotton-Cs/Ag NPs fabrics. Peaks of C and O were detected on the cotton fabric sample (Fig. 3a). A strong peak of Au at ca. 2 keV was related to gold coating of the fabrics. For the cotton-Ag sample, a new peak appeared at ca. 3 keV which could be attributed to the silver signal of AgNPs (Fig. 3b). Another new peak formed at ca. 1.7 keV was observed for cotton-Cs-OSi/AgNPs, which is attributed to silicon atoms. It was observed that the silver content increases with the addition of organosilica.

In agreement with EDX analysis, the ICP data show that the cotton samples treated with Cs-OSH/AgNPs solution have higher silver content than the one treated with Cs/Ag NPs solution. The silver content of fabrics increased from 2.45 g kg^−1^ for Co-Cs/Ag NPs sample to 4.12 g kg^−1^ for the Co-Cs-OSH/Ag NPs sample.

### Washing stability

**Fig. 4:**
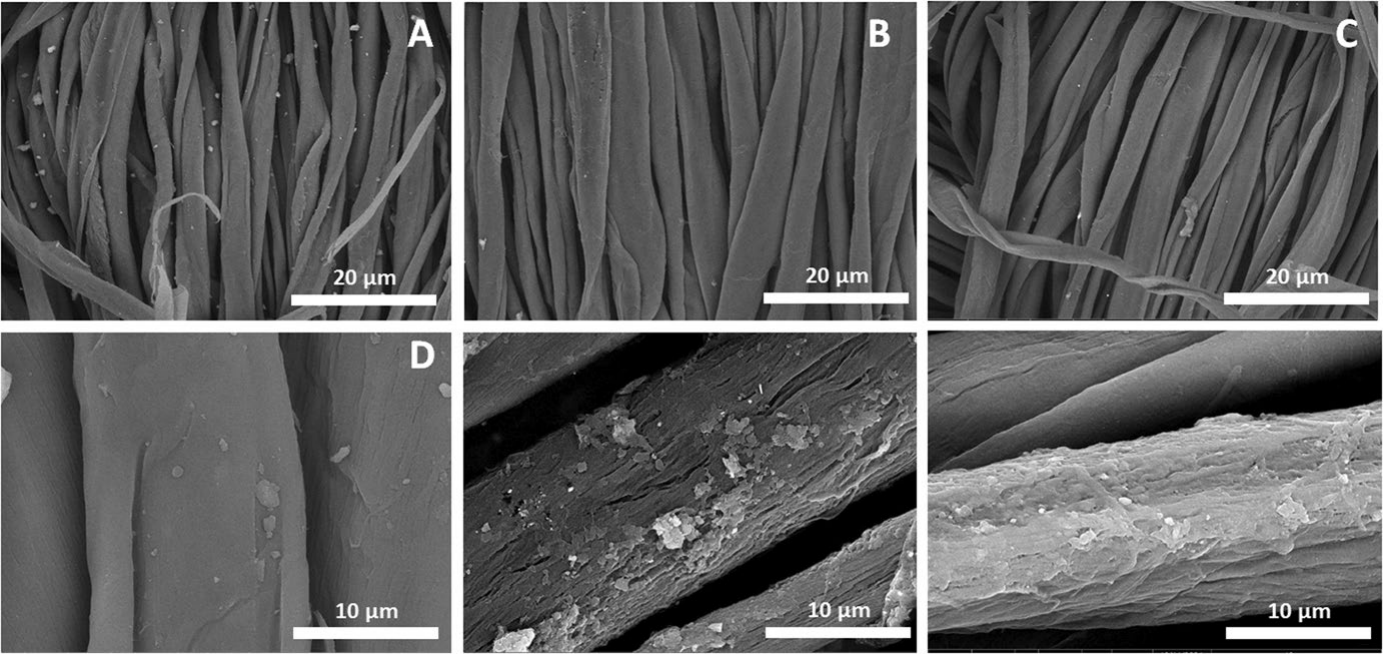
The SEM images of the cotton fabric after 10 times washing at different magnifications

Washing stability represents a key factor in the lack of commercially successful antimicrobial textile products. In addition, the need for long-term and sustained release of antimicrobial agent requires the textile product to be as robust as possible to the chemical detergent and the mechanical stretching resulting from the cleaning processes. The washing stability test confirms the stability of cellulose fiber treated with chitosan–organosilica hybrid compared to chitosan-treated ones. As shown in Fig. 4, it is clearly seen that cotton treated with Cs-OSH/Ag NPs exhibited better surface stability compared to cotton-treated Cs/Ag NPs after washing which could be attributed to stability of Cs-OSH and relatively hydrophobicity resulting from organosilica.

### Antimicrobial activity

**Fig. 5:**
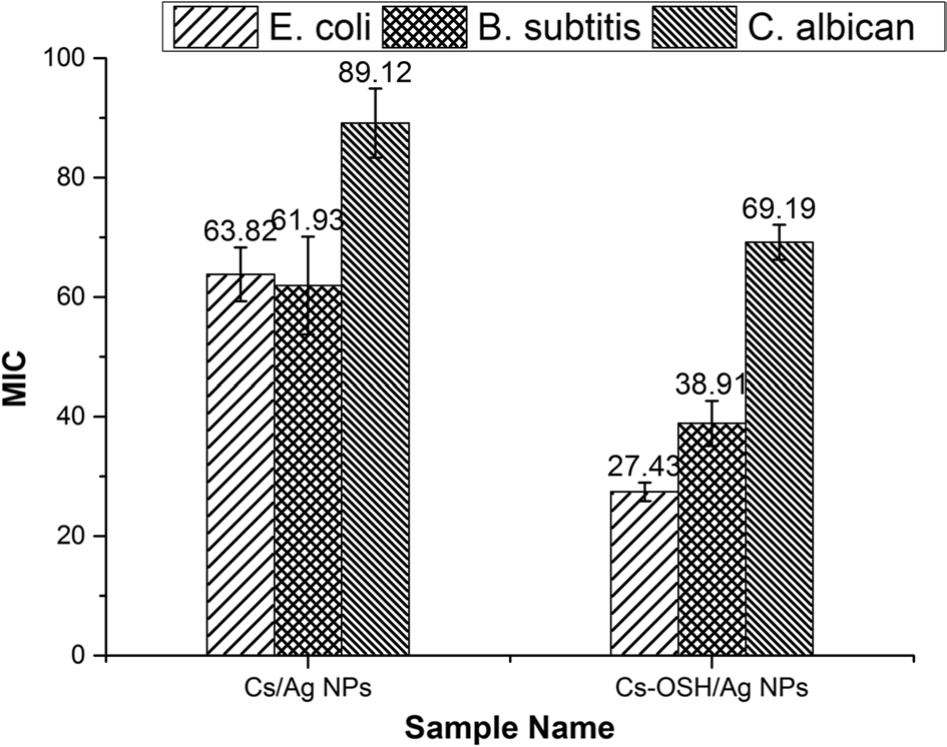
The minimum inhibitory concentrations (MIC) of as-prepared silver nanoparticles against different pathogen isolates (µg/mL). The data were evaluated as mean values ± SD for three experiments

Silver-based materials have been used in numerous application fields due to their unique chemical and physical properties, as well as antimicrobial activity, which allow them to exert various activities, particularly in biomedical devices, pharmaceuticals, food packing and textile applications. Consequently, several factors include particle size and morphology, capping agent and used reducing agent, which are highlighted to affect the antimicrobial activity and toxicity of AgNPs. In the current study, the antimicrobial activity of Ag NPs colloidal solutions synthesized in Cs and Cs-OSH hybrid system was assessed against three different pathogen strains. The results revealed that the Cs/AgNPs sample exhibited good antimicrobial activity toward *E. coli*, *B. subtilis* and *C. albicans*, but it is lower than Cs-OSH/Ag NPs samples. Individually, different concentrations of AgNPs colloidal solutions were tested for antimicrobial activity to detect the minimum inhibitory concentration as shown in Fig. 5. The results demonstrated that MIC of Cs/Ag NPs was 63.82 ± 4.5 µg/mL against *E. coli*, 61.93 ± 8.2 µg/mL for *B. subtilis * and 89.12 ± 5.8 µg/mL toward *C. albicans.* On the other side, the MIC result of Cs-OSH/Ag NPs sample was 27.43 ± 1.6, 38.91 ± 3.8 and 69.19 ± 2.9 µg/mL against *E. coli,* and *B. subtilis* and *C. albicans*, respectively. Notably, the results from the MIC test reveal a high level of antimicrobial activity for the AgNP colloidal solution against *E. coli* with MIC (27.43 μg/ml determined for the Cs-OSH/Ag NPs sample). It was also clear that *C. albicans* appears to be the most resistant to silver nanoparticles.

**Fig. 6:**
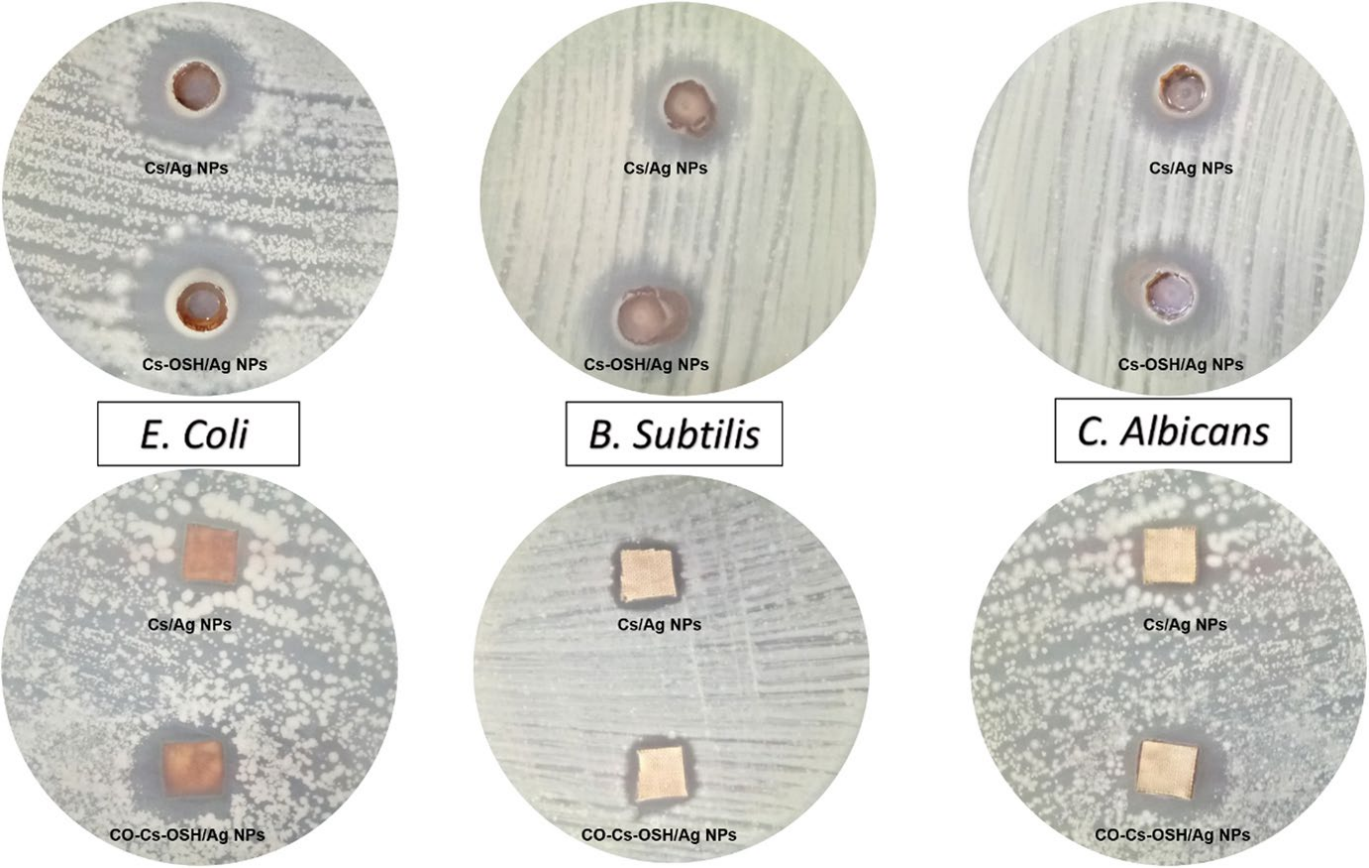
Agar diffusion test of Ag NPs colloidal solutions and cotton-treated samples against * E. coli, B. subtilis* and *C. albicans* after washing 10 times

A comparison between the antimicrobial activity of cotton-treated Cs/Ag NPs and Cs-OSH/AgNPs fabrics toward fungal and bacterial strains was carried out according to inhibition zone. The antimicrobial activity was evaluated for cotton treated samples after washing 10 times using the agar diffusion test, as shown in Fig. 6 and Table 1. The inhibition zones were measured three times in several directions and the results were averaged.

**Table 1:** Antibacterial activity expressed as inhibition zone (mm ± S.D.) after washing 10 times

Sample	Washing cycles	Inhibition zone (mm ± S.D.)		
		*E. coli*	*B. subtilis*	*C. albicans*
Co-Cs/Ag NPs	0	13 ± 3.59	11 ± 2.10	14 ± 3.91
Co-Cs-OSH/Ag NPs	0	15 ± 2.73	10 ± 1.75	17 ± 0.79
Co-Cs/Ag NPs	10	10 ± 0.98	7 ± 1.5	7 ± 1.22
Co-Cs-OSH/Ag NPs	10	13 ± 3.8	9 ± 0.83	15 ± 0.79

The results of the diffusion test are summarized in Table 1, and the images of the antibacterial tests are shown in Fig. 6. It was obvious that the presence of organosilica molecules on cotton surface improved the stability of Cs-AgNPs and significantly increases the antimicrobial activity.

## Discussion

Today, controlling the spread of infectious diseases caused by either bacteria or viruses is a global public healthcare threat and development impendence. Antimicrobial resistance textiles have recently attracted a great attention to fight infectious diseases transmission in medical and health care environments. It is well known that the surface features of natural or synthetic textile provide appropriated environment for bacterial adhesion and growth. Therefore, a wide range of nanoparticles with various structures are applied to impart an antimicrobial potential to textiles. Nowadays, AgNPs are increasingly prevalent in biomedical filed because of its low cellular toxicity and unique physiochemical features and powerful antimicrobial activity along with antiviral activity which represents a major challenge today based on corona virus epidemic. Indeed, numerous studies have reported that chitosan mediates the formation of silver nanoparticles (Ag NPs) via transferring electrons to silver ions as an electron-rich organic macromolecule [33–35]. Comparative studies showed that chitosan is typically used as a capping agent to prevent the aggregation of AgNPs, instead of a potential reducing agent [36]. The current results reported the formation of AgNPs via in situ reduction of silver nitrate in chitosan or chitosan–organosilica matrices using ascorbic acid (as a reducing agent), in which highly stable colloidal solutions of spherical AgNPs of nanosize diameter (less than 20 nm) were prepared.

**Fig. 7:**
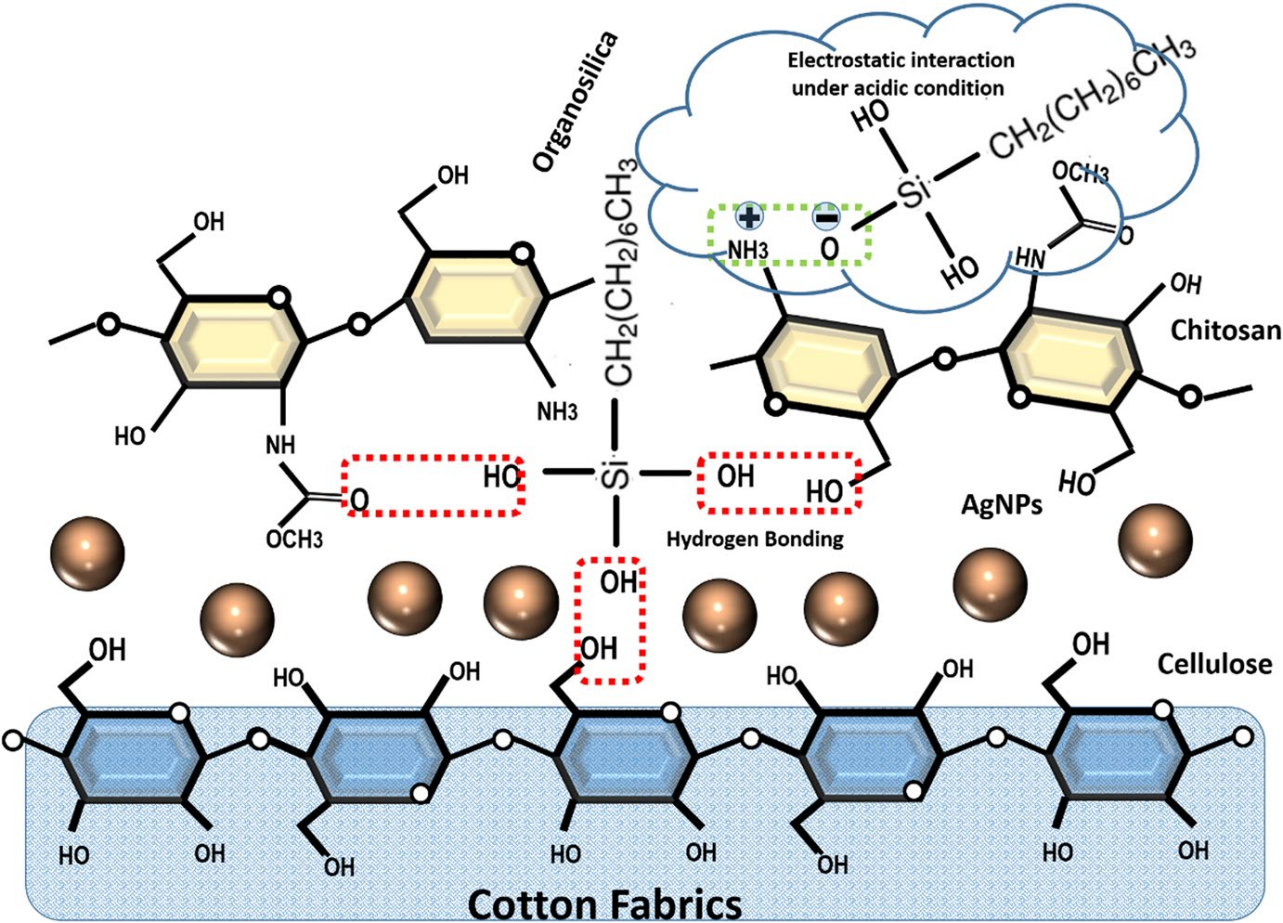
Possible interaction among chitosan, organosilica and cellulose fiber of cotton fabrics

Treatment of cotton fabrics with antimicrobial agents has become a popular approach to produce high performance value-added multifunctional textiles. The synthesized chitosan–organosilica composite belongs to a hybrid material of class I, where chitosan and organosilica hybrid is formed through non-covalent bonds as follows [34, 38]: the hydrogen bonds between the primary amine (–NH_2_), acetamide (–CH_3_CONH_2_) or secondary hydroxyl groups (–CH_2_OH_2_) of the biopolymer and silanol groups(–Si–OH) of silica or electrostatic forces between protonated amino groups (–NH_3_^+^), which act as excellent hydrogen-bonding partners under acidic conditions, and dissociated hydroxyl groups (Si–O^−^). Remarkably, cotton fabrics are made of cellulose, which is a polyhydroxy polysaccharide; therefore, the hydroxyl groups of cellulose saccharide units could also form hydrogen bonds or get into the condensation reaction with silanol groups of organosilica, as shown in Fig. 7.

The washing stability test confirms the stability of cellulose fiber treated with chitosan–organosilica hybrid compared to chitosan-treated ones. In addition, the antibacterial experiments reveal that the antibacterial effect of AgNPs colloidal solution increased with the addition of organosilica. The antibacterial effect of AgNPs was more potent for Cs-OSH sample than that of Cs sample. In general, the antibacterial effect was more effective against *E. coli* (gram-negative bacteria) than *B. subtilis* (gram-positive organisms) as reported before [40].

## Conclusion

Highly concentrated stable colloidal solutions of silver nanoparticles were successfully prepared through eco-friendly via direct reduction of silver nitrate salt using ascorbic acid in chitosan matrix with and without organosilica. TEM observation indicated that AgNP particle size of synthesized in chitosan–organosilica samples was approximately twice higher in case of chitosan. In addition, the chitosan–organosilica hybrid was found to be a prospective adhesive matrix to support AgNPs binding to the fiber of cotton fabrics. SEM images of silver‐treated fabric samples showed uniform particle distribution on the surface of cotton fabric. EDX analysis confirms the presence of silver particles on the fabric surface. Antimicrobial studies exhibited clear inhibition zones on both the treated samples with higher activity against *E. coli*. These results demonstrate that chitosan–organosilica composite is a promising material for improving stability of AgNP on cotton fabric surface for biocide advanced biocide textiles development.
